# Immunologgical self-tolerance in allophenic and embryo-aggregated mice

**DOI:** 10.1186/1742-4682-7-38

**Published:** 2010-09-20

**Authors:** Richmond T Prehn, Liisa M Prehn

**Affiliations:** 1 Dept of Pathology University of Washington 5433 South Hudson St. Seattle,WA, 98118 USA

## Abstract

Allophenic mice, supposedly containing almost equal numbers of cells derived from embryos of mouse strains C57Bl and FVB, were shown in a recent paper to grow the B16 melanoma, a long transplanted tumor of C57Bl origin, much better than did mice of either the parental C57Bl strain or the C57Bl × FVB F1 hybrid. Mice containing smaller proportions of C57Bl cells rejected the tumor. A reconsideration of these suprising data, in light of the current literature, suggests that the better growth of the tumor in the 50-50% allophenics than in the C57Bl parental strain was almost certainly caused by the tumor stimulation engendered by a weak anti-C57Bl immune reaction in the overtly healthy allophenic mice.

## Background

The allophenic mouse, as described by Mintz and Silvers [1], resulted from allowing two embryos of different inbred strains to fuse *in vitro *and then, upon implantation into a surrogate mother, grow into an adult. The adult allophenic mice were of normal size and had no obvious autoimmune problems. In the recent study by Wagner *et al*., that is the subject of the present paper, allophenic mice appeared overtly to be as immunologically tolerant to self as were inbred mice of either of the parental strains [2]. In the particular study by Wagner *et al*., one of the parental strains used was C57Bl. In some cases, varying numbers of C57Bl cells, from eight cell-stage embryos, were aggregated with whole FVB strain embryos, also at the eight cell-stage, to create "embryo-aggregated" chimeric mice with varying proportions of C57Bl cells. A rough idea of the the proportion of C57Bl cells surviving in these chimeric adults could be gained by observing the degree of black coloration of the skin. Rather than using grafts of normal C57Bl tissue to test the anti-C57Bl immune capacities of the aggregated and allophenic animals, the authors used as a surrogate the B16 melanoma, a long transplanted tumor that had originated in the C57Bl strain. This resulted in a seemingly remarkable discovery, a discovery that instigated the present paper [2].

It was found that B16 tumors, implanted s.c., grew significantly *faster *in allophenic mice with full chimerism in their skins as compared with the tumor's growth in either syngeneic C57Bl or semi-syngeneic C57Bl × FVB F1 hosts. In contrast, tumor growth was either absent or significantly reduced in embryo-aggregated mice with reduced numbers of or lacking C57Bl-derived cells in their skin, but tolerant to C57Bl tissue in other organs [2]. The authors apparently found no completely satisfactory explanation for these remarkable findings so we offer the following speculations.

## Speculations

We think it very probable that there was little or no specific anti-tumor immunity; in any case, the tumor antigens were a constant throughout the work. Any tumor-specific immune effects were probably overshadowed by a weak allograft immunity, a possibility suggested by the facts that tumor growth paralleled the presence or absence locally of allogeneic C57Bl cells in the aggregated host's tissues; the growth of the C57Bl tumor cells was directly related to the systemic proportion of normal C57Bl cells. That the fully allophenic mice did have some degree of subliminal anti-C57Bl autoimmunity is supported by the work of Wegmann *et al. *which showed that the lymphoid cells of an allophenic mouse reacted positively against either of the parental strains in an *in-vitro *assay [3]. Furthermore, inhibition of the melanoma, when it occurred in the aggregated mice that were deficient in C57Bl cells, did not appear to develop gradually, but appeared to be preexisting. Thus, we assume that tumor specific immunity played no significant role in the observations of Wagner *et al. *[2] It is apparent that the anti-C57Bl allograft immunity was very weak or the mice could probably not have appeared to be healthy.

How is it that, in the work of Wagner *et al*, the C57Bl melanoma grew *better *in fully chimeric allophenic mice than it did in inbred C57Bl parental strain animals or in the appropriate F1 hybrids?

If the anti-C57Bl immune reaction were, counter intuitively, less in the allophenic than in the parental anti-C57Bl, the better tumor growth in the allophenic would need no further explanation. However, among the various embryo-aggregated mice, as the proportion of C57Bl cells increased (and the proportion of FVB cells decreased) the level of immune resistance to the growth of C57Bl cells steadily decreased. One would logically infer that the anti-C57Bl immunity of the parental C57Bl, having absolutely no FVB cells, would be less than the anti-C57Bl immunity in any of the allophenic or embryo-aggregated mice. This being so, why did the C57Bl tumor grow better in the presumptinely more anti-C57Bl reactive allophenic mice than it did in the mice of the C57Bl parental strain?

**Figure 1:**
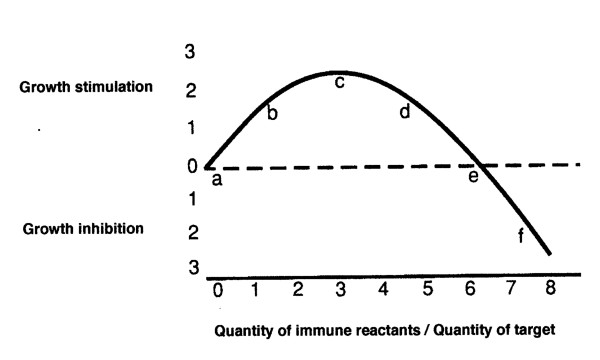
**An idealized representation of the data in citation **[4]** showing the non-linear effect of the relative proportions of immune reactants on the growth of a target tumor**.

That the better growth of C57Bl cells probably occurred in the mouse with greater anti-C57Bl immune reactivity is not without precedent. We suggest that the B16 tumor grew better in the fully allophenic mice than it did in a syngeneic parent in accord with the hormetic shape of the immune response curve (IRC); the IRC relates the stimulation or inhibition of growth to the quantitative size of the specific immune reaction [4]. In general, a weaker immunity has been shown to stimulate tumor growth while a larger quantity of the same immune reactants may be inhibitory [5]. The IRC describes the quantatative relationship between the immune reactants and the growth of target cells, it does not explain why it happens. (See figure 1, -the IRC).

The curve (IRC) is largely based upon observations made with tumor antigens; we think, without direct evidence, that it is probably also applicable to normal allo-antgens. The possible existance of a phenomenon of stimulated growth by a weak immune reaction was first suggested by the possibility that a weak immune reaction to a fetus may be a benefit to fetal survival [6]. Subsequently, the possibility of the phenomenon of immunostimulation of growth was tested directly using Winn tests; a specific number of tumor cells was mixed with various numbers of syngeneic spleen cells harvested from mice that had either bourne or not bourne the specific tumor [4]. The tumor cell/spleen cell mixures were innoculated s.c. into syngeneic mice that had, shortly before, beem radiated and thymectomized in order to prevent, as far as possible, host contributions to any observed immunological effect. The results were clear. The relationship of the quantity of immune reactants to tumor growth was not linear; small proportions of immune spleen cells stimulated tumor growth while larger proportions were inhibitory. An idealized portrayal of this result is shown in figure1. Numerous other titrations, similar in principle, were performed by us as well as by others both *in vitro *and *in vivo *with similar results [5]. It seems particularly evident from titrations done with specific antibody that one and the same immune reactant can probably be stimulatory or inhibitory depending only upon dosage [7].

In addition to the titrations described above, the phenomenon of immunostimulation of tumor growth was further explored indirectly by observations of other types. For example, in two large studies, each confirming the other, it was observed in mouse carcinogenesis studies with 3-methylcholanthrene that the tumors with the shortest latences had intermediate levels of immunogenicity. Thus, the most conducive immune-reaction for rapidity of tumor growth was not minimal; it was apparently a significantly positive, albeit low, reaction [8]. Other studies consistent with the immunostimulation hypothesis are outlined in [5].

In view of all this evidence, we feel that the non-linear immune reaction curve (IRC) relating the quantity of immune reactants to the stimulation or inhibition of tumor growth is generally correct and we feel quite comfortable in using it to help understand the growth behavior of target tissues in the allophenic mice. Thus, we suggest that the weak allograft immunity in the fully allophenic mice produced a stimulatory reaction in the B16 melanoma compared to a lack of an immune reaction in the parental strain. (Compare, in figure 1, 'a', the null reaction in the parental pure strain, with 'b' - 'd', the suggested weak stimulatory reaction to tumor in the fully allophenic mice). In contrast to the full allophenics, those with fewer C57Bl cells often refused to grow the tumor [2]; these mice apparently had a higher degree of anti-C57Bl immunity and fell upon an inhibitory part of the IRC (to the right of 'e').

In two cases, tumors that had failed to grow in the skin of an aggregated chimera deficient in C57Bl cells were subsequently found to have grown well in underlying internal organs [2]. This observation suggests, assuming an accurately aimed tumor inoculum, that some unknown attribute of the skin increased the effectiveness of immunity in that organ while the reaction elsewhere was weaker and thus still in the stimulatory range. This observation further suggests that the anti-C57Bl immunity, probably by both positive and negative selection, may have influenced the organ distribution of the normal C57Bl cells in both the fully allophenic and the embryo aggregated mice.

This analysis unfortunately sheds little light upon the nature of the mechanisms that serve to keep autoimmunity to a harmless level. The embryo-aggregated mice produced by Wagner *et al. *[2] do strongly suggest that the low levels of autoimmunity sufficient to produce tumor stimulation are probably relatively harmless to non-neoplastic cells with shared immunogenic specificities [2]. These mice also serve to suggest what had been assumed, but not previously demonstrated; immunity to normal tissue allo-antigens apparently conforms to an IRC with a non-linear shape much like the curve followed by tumor-specific antigens. However, there appears to be a differential between normal and tumor cells in that normal cells appear to respond to a small immune reaction with less obvious proliferation than do tumor cells and perhaps with a lessened inhibition by somewhat larger immune reactions.

The difference between the degree of self-tolerance in a fully allophenic mouse and in a pure strain parent could be dependent upon the fact that the parent and the F1 mice both have a full load of immunogenic cells while the fully allophenic mouse has only half as much, consisting as it does, of one of the two sets of parental-antigen containing cells. However, it should be noted that the F1, while it presumably has half as many antigens *per *cell, has a full 100% complement of immunogenic cells; apparently it is the proportion of immunogenic cells that counts for the induction of self-tolerance and not the antigenic load *per *cell.

There is much evidence from other systems that suggest that larger antigen dosages favor higher degrees of tolerance [9-13]. as indeed seems to be the case with the embryo-aggregated mice. The work of Wagner *et al. *shows clearly that in the allophenics and embryo aggregated mice the degree of self-tolerance was directly proportional to the degree of chimerism; the fewer the C57Bl cells, the less the self-tolerance and the greater the degree of anti-C57Bl immunity.

In contrast, it is also clear from other studies that tiny antigen dosages, as seen in the "sneaking through phenomenon" [14] and in the relative tolerance of mice to autologous tumors [15,16], can regularly produce a form of immunological tolerance to tumor antigens. How and whether these tolerance phenomena may be related to the self-tolerance mechanisms present in allophenics is not clear, especially since the latter are engendered in embryos rather than in adult antigen recipients.

## Conclusions

In sum, from the work of Wagner and colleagues the following conclusions seem reasonable:

1). Self-tolerance in allophenics is largely a function of the proportion of cells containing the self-antigens rather than the amount of immunogen *per *cell. The greater the proportion of antigen containing cells, the greater the degree of immune self-tolerance. Hence the difference between the full-allophenic and the F1 in stimulating growth of the B16 tumor.

2). Amounts of immune reactants too low to cause overt autoimmunity in allophenic mice may sometimes be sufficient to stimulate the growth of a tumor that has a pertinent allo-antigen. The phenomenon of tumor immunostimulation is thus further supported [17].

3). Normal tissues respond to immunity along curves similar to the IRC except that tumor tissue may be more overtly sensitive to immunostimulation and perhaps also to immune inhibition.

4). Probably none of our speculations bears directly upon the role of regulatory T-cells in self tolerance [18] or on the possibility of immune paralysis [19].

The speculations we have here advanced may well be wrong in whole or in part, but they are internally consistent and are, as we have pointed out, congenial with many other studies. The observations of Wagner *et al. *[2] are of intense interest and fascination; it is certainly satisfying to be able to fit their remarkable results somewhat into the general body of contemporary knowledge.

## Competing interests

The authors declare that they have no competing interests.
